# Circulating clover‐leaf cells presenting in acute‐type adult T‐cell leukemia‐lymphoma

**DOI:** 10.1002/jha2.552

**Published:** 2022-08-27

**Authors:** Yu‐Wei Lin, Yu‐Hsin Hsu, Ming‐Yuan Lee

**Affiliations:** ^1^ Department of Pathology and Laboratory Medicine Koo Foundation Sun Yat‐Sen Cancer Center Taipei Taiwan

1

A 63‐year‐old woman had a mass on the left neck a month ago. She had the lymph node excised at outside hospital and the initial pathological diagnosis was peripheral T‐cell lymphoma. In order of diagnosis confirmation and following treatment, the patient was referred to hematologist. Positron emission tomography–computed tomography scan and bone marrow (BM) studies were assigned by the hematologist for disease staging. Routine complete blood count with differential revealed leukocytosis (17.8 × 10^3^/µl) and thrombocytosis (423 × 10^3^/µl) with atypical lymphocytes of 36%, and serum lactate dehydrogenase was elevated (229 U/L). Peripheral blood film showed a spectrum of atypical lymphocytes characterized by cleaved, lobulated, and clover leaf‐like nuclei, and basophilic cytoplasm with approximately twice the size of an erythrocyte, namely clover‐leaf cells (Figure 1A,B). The positivity for anti‐human T‐cell lymphotropic virus (HTLV)‐1/2 antibody (103.07 S/CO), which was analyzed by chemiluminescent microparticle immunoassay, indicated previous infection of HTLV.

**FIGURE 1:**
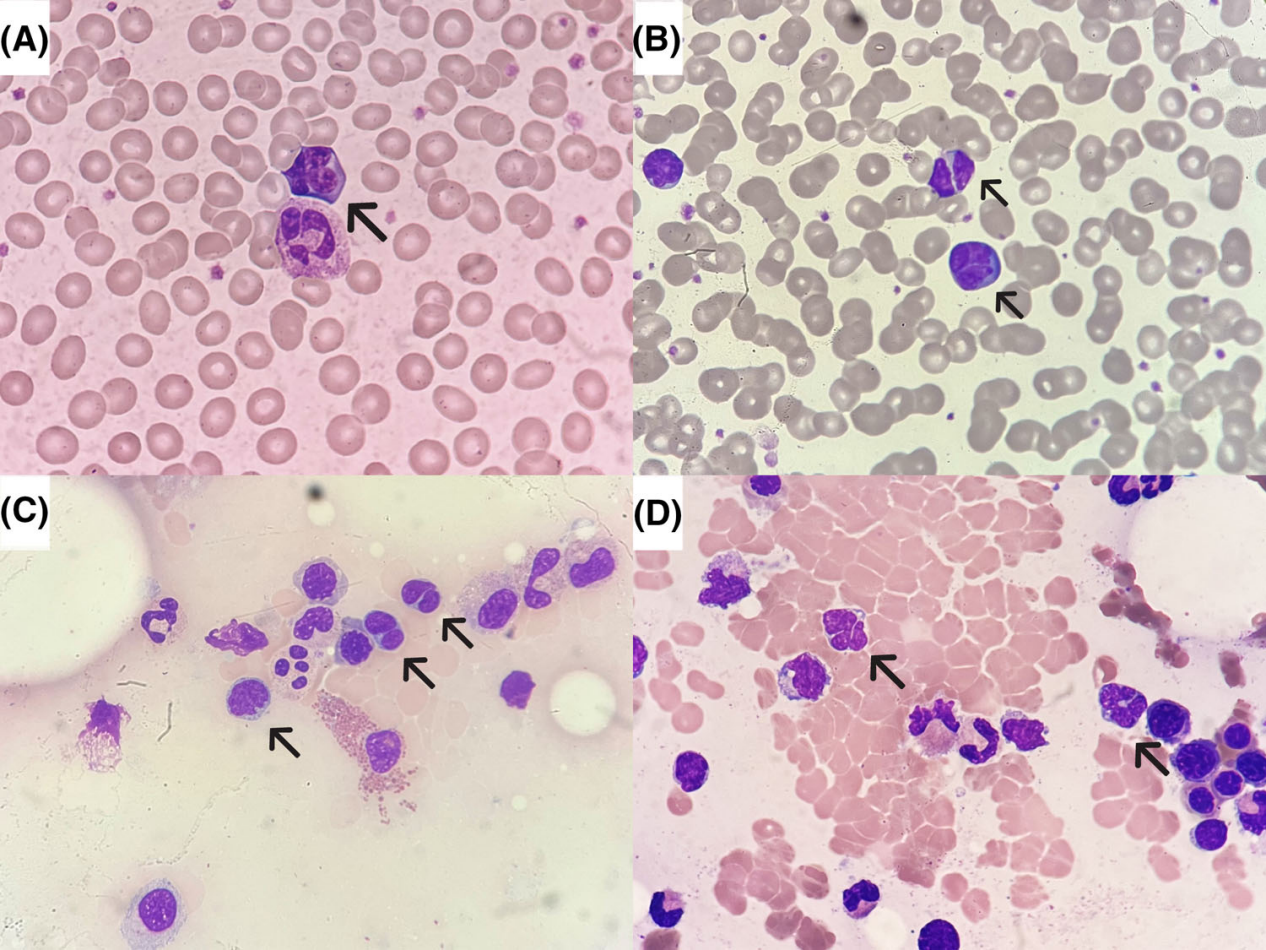
Clover‐leaf cells in peripheral blood film and bone marrow (BM) smears: (A and B) peripheral blood film showed clover‐leaf cells; (C and D) BM smears demonstrated suspected infiltration of leukemic cells (Wright‐Giemsa stain, 1000×).

BM smears demonstrated few scattered atypical lymphocytes (Figure 1C,D). Few small mature T cells expressed CD8 positivity in BM biopsy. Nevertheless, immunohistochemical studies of BM were not contributory. Flow cytometry study of BM aspirates showed presence of a monoclonal T‐cell population of 14.8% (CD3+, CD4+, CD25±, CD45+) with aberrant loss of CD7, which was compatible with T‐cell lymphoma involvement. Her imaging studies showed focal increased uptake of bilateral lungs, left buttock, paranasal sinuses, bilateral iliac bones, and adjacent soft tissue. Acute‐type adult T‐cell leukemia–lymphoma (ATLL) with multifocal involvement was diagnosed based on laboratory results, pathological findings, and imaging studies. She received chemotherapy but passed away due to disease progression.

We emphasized on a case of ATLL revealing clover‐leaf cells in peripheral and BM with history of HTLV infection. Since ATLL is caused by HTLV‐1 infection, high incidence of it occurs in HTLV‐endemic regions, including southern western Japan, Caribbean basin, and central Africa [1]. Incidence of ATLL, however, is increasing in non‐endemic regions worldwide due to population migration [2]. Notably, a confirmatory test of HTLV‐1 such as western blot should be applied due to high false‐positive rate of enzyme‐based immunoassays in non‐endemic areas for HTLV‐1 [3]. Though our case was limited by lack of confirmatory testing for HTLV‐1, high S/CO value was associated with higher diagnostic reliability of HTLV infection as reported previously [3]. When medical laboratory professionals identify clover‐leaf cells on the blood smear, we may recommend physicians to apply additional serology test of HTLV for differential diagnosis of ATLL.

## AUTHOR CONTRIBUTIONS

Yu‐Wei Lin wrote the manuscript and took the pictures of blood smears and bone marrow smears. Yu‐Hsin Hsu and Ming‐Yuan Lee made the revisions.

## CONFLICTS OF INTEREST

The authors declare they have no conflicts of interest and no financial relationships at present or within the previous 3 years with any organizations having an interest in the submitted article.

## FUNDING INFORMATION

All authors have declared that no funding was received from any organization for the submitted article.

2

## ETHICS STATEMENT

The submitted article was designated as exempt from review of IRB (Koo Foundation Sun Yat‐Sen Cancer Center) by the chairman.

## PATIENT CONSENT STATEMENT

No patient identifiable images or data have been included in the article. Verbal informed consent was obtained from the patient's next‐of‐kin.

## Data Availability

Data sharing was not applicable to this article since no datasets were generated or analyzed in the submitted article.
